# Supplementary material for: Wax Composition of Serbian Dianthus spp. (Caryophyllaceae): Identification of New Metabolites and Chemotaxonomic Implications
Source: Plants (Basel). 2023 May 24;12(11):2094. doi: 10.3390/plants12112094 (PMC10255586; doi:10.3390/plants12112094)
Supplement: Supplementary file 1 [file plants-12-02094-s001.zip › plants-2402794-supplementary.pdf]

Supplementary Material

Wax Composition of Serbian *Dianthus* spp. (Caryophyllaceae): Identification of New Metabolites and Chemotaxonomic Implications

Marko Z. Mladenović [1], Milenko N. Ristić [2], Andrija I. Bogdanović [3], Novica R. Ristić [2], Fabio Boylan [4,*] and Niko S. Radulović [1,*]

[1] Department of Chemistry, Faculty of Sciences and Mathematics, University of Niš, Višegradska 33,
18000 Niš, Serbia; markohem87@gmail.com
[2] Faculty of Natural Science and Mathematics, University of Priština, Lole Ribara 29, 38220 Kosovska Mitrovica, Serbia; milenko.ristic@pr.ac.rs (M.N.R.); novica.ristic@pr.ac.rs (N.R.R.)
[3] Department of Biology and Ecology, Faculty of Sciences and Mathematics, University of Niš, Višegradska 33, 18000 Niš, Serbia; andrijab8@hotmail.com
[4] School of Pharmacy and Pharmaceutical Sciences, Panoz Institute, and Trinity Biomedical Sciences Institute, Trinity College Dublin, D02 PN40 Dublin, Ireland
* Correspondence: fabio.boylan@tcd.ie (F.B.); nikoradulovic@yahoo.com (N.S.R.); Tel.: +353-1-896-4154 (F.B.); Tel.: +381-18-533-015 (N.S.R.); Fax: +381-18-533-014 (N.S.R.)

Table of contents:

Table S1. The identified constituents detected only after the silylation procedure and their abundance.
Table S2. Percentage composition of wax constituents in the chromatographic fractions of *D. superbus* flowers washings.
Figure S1. Typical total ion current (TIC) chromatogram of diethyl ether extract of *Dianthus petraeus* flowers (sample **5a** in Table 1).
Figure S2. Mass spectrum of **[A]** eicosyl tiglate, **[B]** eicosyl senecioate, and **[C]** eicosyl angelate.
Figure S3. ^{1}H NMR spectrum of eicosyl tiglate recorded in $CDCl_3$.
Figure S4. ^{13}C NMR spectrum of eicosyl tiglate recorded in $CDCl_3$.
Figure S5. DEPT 90 and DEPT 135 spectra of eicosyl tiglate recorded in $CDCl_3$.
Figure S6. Proton-coupled ^{13}C NMR spectrum of eicosyl tiglate recorded in $CDCl_3$.
Figure S7. HSQC spectrum of eicosyl tiglate recorded in $CDCl_3$.
Figure S8. HMBC spectrum of eicosyl tiglate recorded in $CDCl_3$.
Figure S9. Part of the GC chromatogram before (**A**) and after (**B**) derivatization of the crude wax sample with hydrazine.

Figure S10. Mass spectra of nonacosane-12,14-dione (**A**), hentriacontane-14,16-dione (**B**), and tritriacontane-16,18-dione (**C**).

Figure S11. Mass spectra of pyrazole derivatives obtained from nonacosane-12,14-dione (**A**), hentriacontane-14,16-dione (**B**), and tritriacontane-16,18-dione (**C**).

Figure S12. Part of the GC chromatogram before (**A**) and after (**B**) silylation of the crude wax sample.

Figure S13. Mass spectra of mixtures of silylated enol forms of nonacosane-12,14-dione (**A**), hentriacontane-14,16-dione (**B**), and tritriacontane-16,18-dione (**C**).

Figure S14. Mass spectra of triacontane-14,16-dione (**A**), dotriacontane-14,16-dione (**B**), tetratriacontane-16,18-dione (**C**), and pentatriacontane-16,18-dione (**D**).

Figure S15. Patrial ion current chromatogram (PIC, ions at: **A**: *m/z* 96 and 109, **B**: *m/z* 292 and 305, and **C**: *m/z* 264 and 277) of derivatized chromatographic fraction of *D. superbus* flowers washings.

Figure S16. **A** - Part of the chromatogram (ca. 48 – 51 min) of the silylated wax sample; **B** - mass spectrum of unknown detected constituent at Rt = 49.15 min; **C** - mass spectrum of 1-(trimethylsilyloxy)dotriacontane detected at Rt = 49.56 min.

Figure S17. Dendrogram of AHC obtained by agglomerative hierarchical clustering using the transformed sums of constituent classes and representing the chemical-composition dissimilarity relationships of 16 wax samples (observations) of 7 different *Dianthus* taxa (15 samples) and one sample of *P. prolifera*. As a dissimilarity metric, the Euclidian distance was used (dissimilarity within the interval [0, 5800], using Ward's method as an aggregation criterion). Two statistically different groups of oils were found (**C1**–**C2**).

Figure S18. Dendrogram of PCA obtained by principal component analysis using the transformed sums of constituent classes and representing the chemical-composition dissimilarity relationships of 16 wax samples (observations) of 7 different *Dianthus* taxa (15 samples) and one sample of *P. prolifera*.

Figure S19. Esterification (A; DCC - *N*,*N*′-dicyclohexylcarbodiimide, DMAP - 4-(dimethylamino)pyridine), silylation (B), dimethyl disulfide (DMDS) derivatization (C), and the synthesis of pyrazoles (D).

Table S1. The identified constituents detected only after the silylation procedure and their abundance.

RI[a]	Compound	Samples[b]															ID[c]
		**1a**	**1b**	**1c**	**1d**	**2a**	**2b**	**2c**	**3**	**4**	**5a**	**5b**	**5c**	**6a**	**6b**	**7**	
1007	3-Methyl-2-butenoic acid	tr	tr													tr	MS, RI
1011	(*E*)-2-Methyl-2-butenoic acid															tr	MS, RI
1071	Hexanoic acid	0.1	tr											tr			MS, RI
1167	Heptanoic acid	0.3	tr	tr													MS, RI
1247	Benzoic acid	tr				tr	0.4	tr						0.3	tr		MS, RI
1264	Octanoic acid	tr												tr			MS, RI
1285	Glycerol	0.1	tr				0.1	tr						0.1			MS, RI
1301	Decan-2-ol														tr		MS, RI
1303	Benzeneacetic acid													tr			MS, RI
1319	Butanedioic acid						0.2	tr						tr	tr		MS, RI
1362	Nonanoic acid	0.2	tr	tr					tr					tr	tr	tr	MS, RI
1375	Decan-1-ol													tr			MS, RI
1459	Decanoic acid	tr	tr	tr						tr	tr			0.2	tr	tr	MS, RI
1463	2-Methoxybenzoic acid													0.3			MS, RI
1509	4-Hydroxybenzyl alcohol													tr			MS, RI
1524	2-Hydroxybenzoic acid													tr	tr		MS, RI
1546	3-Phenyl-2-propenoic acid													tr			MS, RI
1557	Undecanoic acid	tr							tr	tr				tr			MS, RI
1568	4-Hydroxyphenylethanol						tr							tr	0.1		MS, RI
1579	2-Hydroxy-3-phenylpropionic acid					tr											MS, RI
1592	3-Hydroxy-3-methylglutaric acid						0.3										MS, RI

1596	Tridecan-2-ol	tr	tr														MS, RI
1624	4'-Hydroxy-3'-methoxyacetophenone													0.2			MS, RI
1627	4-Hydroxybenzoic acid			tr		tr	tr	0.1						0.2	tr		MS, RI
1654	Dodecanoic acid	tr	tr								tr	tr		0.1		tr	MS, RI
1694	Tetradecan-2-ol									tr							MS, RI
1706	Octanedioic acid										tr						MS, RI
1719	3,4-Dimethoxybenzoic acid													tr			MS, RI
1751	Tridecanoic acid										tr			tr	tr		MS, RI
1752	Xylitol	0.1	tr														MS, RI
1755	4-Hydroxyphenylpropionic acid					tr					tr						MS, RI
1792	Pentadecan-2-ol	tr	tr								tr			tr	0.1		MS
1803	Nonanedioic acid	tr	tr	tr			0.4	tr			tr	tr		tr	tr		MS, RI
1832	3,4-Dihydroxybenzoic acid					tr	tr	tr	tr					0.2	0.1		MS, RI
1849	Tetradecanoic acid	tr	0.1			tr	tr	0.1		tr	0.1	0.1	tr	tr	0.2	tr	MS, RI
1889	Hexadecan-2-ol													tr			MS, RI
1897	3-(4-Hydroxy-3-methoxyphenyl)propionic acid	tr		0.5		2.6	0.5	tr	tr	tr	tr			0.5	0.2		MS, RI
1909	Syringic acid													tr	0.1		MS, RI
1910	13-Methyltetradecanoic acid							tr			tr						MS
1919	12-Methyltetradecanoic acid														tr	tr	MS
1944	Cinnamic acid					0.7	0.9	0.1	tr					0.1	tr		MS, RI
1947	Pentadecanoic acid	tr	tr	tr		tr	0.1	0.1		0.1	tr	tr		tr	tr	tr	MS, RI
1966	D-Mannitol	0.1															MS, RI
1986	Heptadecan-2-ol	tr	tr								tr			tr	0.2	tr	MS
1990	Undecanedioic acid	tr		tr				tr			tr						MS, RI

2016	*cis*-9-Hexadecenoic acid	tr					tr	0.1			tr	0.1			0.1		MS, RI
2018	13-Methylpentadecanoic acid		tr													tr	MS
2084	Octadecan-2-ol		tr														MS
2084	2-Methylhexadecanoic acid															tr	MS
2085	(*E*)-Isoferulic acid	tr				0.6	0.4	tr	tr								MS, RI
2099	Ferulic acid													0.1	0.1		MS, RI
2117	14-Methylhexadecanoic acid	tr	tr				tr					tr		tr		tr	MS
2127	Caffeic acid					tr											MS, RI
2181	Nonadecan-2-ol										tr						MS
2188	Hexadecane-1,2-diol					0.1	0.1	0.1									MS, RI
2205	16-Methylheptadecanoic acid	0.1												tr		tr	MS
2216	Oleic acid	0.4	0.1	0.1		0.6	0.1	1.9	tr	0.1	0.2	0.2	15.1	0.5	tr	1.1	MS, RI
2221	(*E*)-9-Octadecenoic acid	tr	tr											tr			MS, RI
2242	Octadecanoic acid	0.5	0.2	0.6	tr	0.2	0.4	0.7	tr	1.1	0.2	0.7	7.3	0.3	0.3	1.1	MS, RI
2278	Eicosan-2-ol														tr		MS
2315	16-Methyloctadecanoic acid			tr			tr									tr	MS, RI
2341	Nonadecanoic acid	0.1	tr	0.2			tr		tr		0.1	tr		0.2	0.1	tr	MS, RI
2375	Heneicosan-2-ol		tr									tr		tr			MS
2408	(*Z*,*Z*)-11,14-Eicosadienoic acid	0.1		0.2		0.1	0.3	0.1			0.1			tr	tr		MS, RI
2410	*cis*-13-Eicosenoic acid			tr			tr	0.2			0.1				tr		MS, RI
2439	Eicosanoic acid	0.5	0.2	0.9	tr	0.3	0.3	0.5	0.1	tr	0.1	0.2	tr	tr	0.2	0.3	MS, RI
2490	Hexadecanedioic acid		tr														MS, RI
2511	18-Methyleicosanoic acid						tr										MS, RI
2538	Heneicosanoic acid	0.3	0.1	0.6		0.1	0.2	0.2	0.1	0.2	0.5	0.1		0.5	0.1	tr	MS, RI

2572	Tricosan-2-ol	tr									tr	tr		tr	tr		MS
2598	1-Monopalmitoylglycerol					0.6	1.2	0.3			tr	tr		0.8	0.1		MS, RI
2608	*cis*-13-Docosenoic acid										tr					tr	MS, RI
2610	19-Methylheneicosanoic acid	tr															MS
2637	Docosanoic acid	1.3	0.6	1.8	0.4	0.7	1.3	1.7	0.8	1.1	0.4	0.6		0.2	0.3	0.5	MS, RI
2646	Tricosan-1-ol	0.2	0.1	0.3		tr	0.5	0.2			0.2			0.3		tr	MS, RI
2670	Tetracosan-2-ol														tr		MS
2699	2-Monolinoleoylglycerol					0.3											MS, RI
2736	Tricosanoic acid	0.3	0.1	0.6	tr	0.1	0.3	0.6	0.3	0.1	0.2	0.1			tr	tr	MS, RI
2744	Tetracosan-1-ol	3.4	1.2	3.1	1.5	1.1		2.7	1.1	2.3	1.2	0.9	tr	1.8	1.1	3.8	MS, RI
2755	2-Oleoylglycerol					0.6	2.1							tr	0.2		MS, RI
2769	Pentacosan-2-ol	tr	tr	0.6							tr	tr		tr	tr	tr	MS, RI
2791	α-Glyceryl stearate													0.9			MS, RI
2799	22-Methyltricosanoic acid		tr	tr		tr	0.1										MS, RI
2807	21-Methyltricosanoic acid	tr					tr	tr									MS, RI
2835	Tetracosanoic acid	3.5	1.6	4.7	0.8	1.1	1.6	2.2	1.1	2.0	0.7	1.2		0.4	0.6	1.7	MS, RI
2841	Pentacosan-1-ol	0.3	0.2	0.6							0.2			tr	0.1	0.4	MS, RI
2867	Hexacosan-2-ol		tr														MS
2906	22-Methyltetracosanoic acid			tr			tr	tr	tr								MS, RI
2934	Pentacosanoic acid	0.2	0.2	0.4		0.1	0.7	0.7	0.2	0.1	0.2	0.2		tr	tr	0.2	MS, RI
2965	Heptacosan-2-ol		0.1	0.4							tr	tr			0.2		MS, RI
2997	24-Methylpentacosanoic acid	tr															MS, RI
3033	Hexacosanoic acid	2.0	2.1	1.5	1.2	0.9	1.1	2.3	1.5	4.8	0.5	1.4		0.6	0.4	3.1	MS, RI
3038	Heptacosan-1-ol	0.4	0.3	0.6	0.6			0.5	tr		0.4	0.6		0.2	0.4	0.4	MS, RI

3063	Octacosan-2-ol		tr														MS
3131	Heptacosanoic acid	0.1	tr	tr							tr	tr			tr	tr	MS, RI
3161	Nonacosan-2-ol		tr	tr								tr			tr	tr	MS, RI
3194	26-Methylheptacosanoic acid	tr		tr													MS, RI
3203	25-Methylheptacosanoic acid						tr					tr					MS, RI
3230	Octacosanoic acid	1.6	1.2	2.3	0.4	1.1	1.2	1.6	1.1	1.9	1.6	3.9		0.8	1.1	1.1	MS, RI
3234	Nonacosan-1-ol														0.3	0.4	MS, RI
3328	Nonacosanoic acid		1.9									tr				0.4	MS, RI
3359	Hentriacontan-2-ol											0.4					MS
3427	Triacontanoic acid	tr	tr	0.8	0.5	1.1	1.2	2.3	tr	1.2	tr			tr	3.6	2.1	MS, RI
3491	30-Methylhentriacontan-1-ol										tr						**NEW**
3497	28-Methyltriacontanoic acid	tr				0.2	0.2		tr			tr					MS, RI
3525	Hentriacontanoic acid		tr	1.5	1.2	0.6	0.6	1.5		0.3						tr	MS, RI
3528	Dotriacontan-1-ol	1.5	0.6								0.7	1.0		0.4	0.3	2.9	MS, RI
3623	Dotriacontanoic acid	0.5	2.1	0.2	0.7	0.5	0.2	1.4	0.2	0.1		1.6		tr	1.8	3.0	MS, RI
3687	32-Methyltritriacontan-1-ol	0.2															MS
3726	Tetratriacontan-1-ol	0.5	tr	0.3							tr	0.2			0.1	0.7	MS, RI
3820	Tetratriacontanoic acid		0.1					0.2		tr	0.1	0.1				0.3	MS, RI
3825	Pentatriacontan-1-ol															0.7	MS
3878	Triacontanedioic acid							0.2	tr			tr					MS, RI
3923	Hexatriacontan-1-ol	tr														tr	MS, RI

[a] RI – retention indices on a DB-5 column calculated against a series of co-injected *n*-alkanes for TMS derivatives; [b] Values are means of three individual analyses; tr, trace amounts (< 0.05%); [c] ID = Compound identification: MS, mass spectra matching; RI, retention indices matching with literature data.

Table S2. Percentage composition of wax constituents in the chromatographic fractions of *D. superbus* flowers washings

RI[a]	Compound	Sample 6a[b]							I[c]	ID[d]
		F1	F2	F3	F4	F5	F6	F7		
802	Hexanal				tr				+	MS, RI, CoI
834	2-Methylbutanoic acid							tr	-	MS, RI, CoI, Si
852	(*Z*)-Hex-3-en-1-ol						tr		+	MS, RI, CoI, Si
867	Hexan-1-ol							tr	-	MS, RI, CoI, Si
900	Nonane	tr	tr						+	MS, RI, CoI
959	Benzaldehyde				tr				-	MS, RI, CoI
960	(*Z*)-Hept-2-enal				tr				+	MS, RI, Si
966	Heptan-1-ol							tr	-	MS, RI, CoI
979	6-Methyl-5-hepten-2-one			tr	tr				-	MS, RI
994	2-Pentyl furan							tr	-	MS, RI
1000	Decane	tr							+	MS, RI, CoI
1001	Octanal				tr				-	MS, RI, CoI
1005	(*Z*)-Hex-3-en-1-yl acetate			tr					-	MS, RI
1036	Benzyl alcohol							tr	+	MS, RI, CoI, Si
1047	Phenylacetaldehyde				tr				-	MS, RI
1049	(*E*)-β-Ocimene			0.1					-	MS, RI
1069	Octan-1-ol							tr	+	MS, RI, Si
1100	Undecane	tr							+	MS, RI, CoI
1106	Nonanal				0.1				+	MS, RI, CoI
1120	Maltol						tr	tr	+	MS, RI, Si

1146	Veratrole							tr	-	MS, RI, Si
1190	Dodec-1-ene			tr					+	MS, RI
1200	Dodecane	tr	tr						+	MS, RI, CoI
1206	Decanal			tr	0.1				+	MS, RI, CoI
1274	3-Methyldodecane	tr							+	MS, RI
1300	Tridecane	tr							+	MS, RI, CoI
1309	Undecanal				tr				+	MS, RI
1318	1,2,3-Trimethoxybenzene					tr			-	MS, RI
1340	Methyl *o*-methoxybenzoate			tr					+	MS, RI
1367	(*E*)-Undec-2-enal			tr	tr				+	MS, RI
1373	Undecan-1-ol							tr	-	MS, RI, Si
1374	Butyl benzoate			tr					-	MS, RI
1392	Tetradec-1-ene		tr						-	MS, RI
1400	Tetradecane	tr	tr						+	MS, RI, CoI
1401	Dodecan-2-ol							tr	-	MS, RI, Si
1408	(*Z*)-Caryophyllene	0.1							+	MS, RI
1412	Dodecanal				0.1				-	MS, RI
1414	2-Methylundecan-1-ol							tr	-	MS, RI, Si
1425	(*E*)-Caryophyllene	tr							+	MS, RI, CoI
1431	10-Methylundecan-1-ol							tr	-	MS, RI, Si
1437	9-Methylundecan-1-ol							tr	-	MS, RI, Si
1457	α-Humulene		0.1						+	MS, RI
1476	Dodecan-1-ol							1.2	-	MS, RI, Si
1484	Germacrene D		tr						+	MS, RI, CoI

1494	Apocynin							1.2	+	MS, RI
1498	Tridecan-2-one			tr					-	MS, RI
1500	Pentadecane	tr							+	MS, RI, CoI
1502	(*E*)-Methyl isoeugenol						tr		+	MS, RI, Si
1513	Tridecanal				0.6				+	MS, RI
1527	δ-Cadinene		tr						+	MS, RI
1532	11-Methyldodecan-1-ol							tr	-	MS, RI, Si
1538	10-Methyldodecan-1-ol							tr	-	MS, RI, Si
1538	Phenylethyl pentanoate			tr					-	MS, RI
1579	Tridecan-1-ol							0.4	-	MS, RI, CoI, Si
1579	(*E*)-Dendrolasin							tr	-	MS, RI
1581	Isovanillic acid							tr	+	MS, RI, Si
1593	Caryophyllene oxide				2.7	27.4	tr		+	MS, RI, CoI
1600	Hexadecane	tr	tr						+	MS, RI, CoI
1602	Clovenol						tr		-	MS, RI
1608	3,4,5-Trimethoxybenzaldehyde						tr		-	MS, RI
1616	Tetradecanal				tr				+	MS, RI
1620	Humulene epoxide II				0.1				-	MS, RI
1645	Caryophylla-4(12),8(13)-dien-5-*α*-ol				0.1	1.1	2.4	2.5	-	MS, RI, Si
1648	Caryophylla-4(12),8(13)-dien5-β-ol				0.2	4.1	2.6	10.4	-	MS, RI, Si
1653	(*E*)-Asarone					3.4			-	MS, RI
1667	(*Z*)-14-Hydroxycaryophyllene						2.7	0.5	-	MS, RI
1675	(*E*)-Tetradec-2-enal				tr				-	MS, RI
1681	Tetradecan-1-ol							tr		MS, RI, Si

1682	Caryophylla-3,8(15)-dien-5β-ol						7.0	3.2	-	MS, RI, Si
1689	8-Hydroxyisobornyl isobutanoate							2.9	-	MS, RI
1690	(*E*)-2,3-Dihydrofarnesol							tr	+	MS, RI, Si
1699	Pentadecan-2-one				0.1				+	MS, RI
1700	Heptadecane	tr							+	MS, RI, CoI
1717	Pentadecanal			tr	0.5				+	MS, RI
1723	(2*E*,6*Z*)-2,6-Farnesol							tr	-	MS, RI, Si
1734	(2*Z*,6*E*)-2,6-Farnesol							tr	-	MS, RI, Si
1783	Pentadecan-1-ol							tr	-	MS, RI, Si
1790	Octadec-1-ene		tr						+	MS, RI
1800	Octadecane	tr	tr	tr					+	MS, RI, CoI
1803	2,3-Dihydrofarnesyl acetate				tr				-	MS, RI
1809	Tetradecyl acetate				tr				-	MS, RI
1820	Hexadecanal				0.2				+	MS, RI
1824	Methyl pentadecanoate		tr						-	MS, RI
1839	Neophytadiene (isomer II)				tr				+	MS, RI
1846	Hexahydrofarnesyl acetone				0.1				-	MS, RI
1885	Hexadecan-1-ol				0.2	0.4	4.4	9.5	+	MS, RI, Si
1886	Methyl 14-methylpentadecanoate		tr						-	MS, RI
1900	Nonadecane	tr							+	MS, RI, CoI
1903	Heptadecan-2-one				0.1				+	MS, RI
1922	Heptadecanal			tr	0.2				+	MS, RI
1925	Methyl hexadecanoate		74.1	0.1	0.1				-	MS, RI
1945	Phytol					2.2			-	MS, RI, Si

1950	5-Methylnonadecane	tr							-	MS, RI
1970	Hexadecanoic acid							0.7	+	MS, RI, CoI, Si
1987	Methyl 15-methylhexadecanoate		0.5						-	MS, RI
1990	Eicos-1-ene		tr						-	MS, RI
1995	Methyl 14-methylhexadecanoate		0.4						-	MS, RI
2000	Eicosane	tr	tr	0.1	0.1				+	MS, RI, CoI
2006	Hexadecyl acetate			tr	0.2				+	MS, RI
2024	Octadecanal				0.1				+	MS, RI
2025	Methyl heptadecanoate		1.3						-	MS, RI
2027	(*E*,*E*)-Geranyl linalool					2.6			+	MS, RI
2057	(*Z*,*Z*)-9,12-Octadecadien-1-ol						3.5	11.0	-	MS, RI, Si
2063	2-Methyleicosane	tr							+	MS, RI
2074	(*E*)-Heneicos-10-ene	0.2							+	MS, RI
2089	Octadecan-1-ol							tr	+	MS, RI, CoI, Si
2100	Heneicosane	0.7	tr	tr					+	MS, RI, CoI
2103	Methyl linoleate		tr						+	MS, RI, CoI
2105	γ-Hexadecalactone			tr					+	MS, RI
2105	Nonadecan-2-one				tr				-	MS, RI
2107	Methyl oleate		0.6						-	MS, RI, CoI
2113	(*E*)-Phytol							tr	+	MS, RI, CoI, Si
2127	Nonadecanal			0.1	0.9				+	MS, RI
2128	Methyl octadecanoate		5.4						-	MS, RI, CoI
2143	(*Z*,*Z*)-9,12-Octadecadienoic acid							1.5	+	MS, RI, Si
2149	(*Z*,*Z*,*Z*)-9,12,15-Octadecatrienoic acid							3.4	+	MS, RI, Si

2163	2-Methylheneicosane	tr							+	MS, RI
2173	(*E*)-Docos-10-ene	tr							+	MS, RI
2192	Nonadecan-1-ol							tr	+	MS, RI, Si
2200	Docosane	0.2	tr						+	MS, RI, CoI
2210	Eicosan-2-one				tr				-	MS, RI
2224	Methyl nonadecanoate		0.6						-	MS, RI
2229	Eicosanal				0.4				+	MS, RI
2235	(*Z*)-Tricos-10-ene	0.1							+	MS, RI
2264	2-Methyldocosane	0.2	tr						+	MS, RI
2275	(*E*)-Tricos-10-ene	3.8[e]	tr[e]	tr[e]					+	MS, RI, DMDS
2275	(*E*)-Tricos-9-ene								+	MS, RI, DMDS
2280	(*E*)-Tricos-7-ene	tr							-	MS, RI, DMDS
2292	Tricos-1-ene		tr						+	MS, RI
2296	Eicosan-1-ol							tr	+	MS, RI, Si
2300	Tricosane	5.1	0.7	0.1	0.1				+	MS, RI, CoI
2312	Heneicosan-2-one				tr				-	MS, RI
2315	Tridecyl benzoate			tr					+	MS, RI, CoI
2324	Methyl eicosanoate		tr						-	MS, RI
2332	Heneicosanal			0.9	2.5	1.1			+	MS, RI
2365	2-Methyltricosane	tr							+	MS, RI
2373	3-Methyltricosane	tr							+	MS, RI
2375	(*E*)-Tetracos-9-ene	0.5							-	MS, RI
2382	Hexadecyl hexanoate			tr					+	MS, RI
2398	Heneicosan-1-ol						tr	tr	-	MS, RI, Si

2400	Tetracosane	0.3	tr	tr					+	MS, RI, CoI
2414	Docosan-2-one				tr				+	MS, RI
2418	Tetradecyl benzoate			tr					+	MS, RI, CoI
2424	Methyl heneicosanoate		tr						-	MS, RI
2434	Docosanal			0.1	1.1				+	MS, RI
2435	(*Z*)-Pentacos-10-ene	0.9[e]							+	MS, DMDS
2435	(*Z*)-Pentacos-9-ene								+	MS, DMDS
2440	(2*E*,6*E*)-2,6-Farnesyl benzoate			tr					+	MS, RI, CoI
2464	2-Methyltetracosane	0.4	tr						+	MS, RI
2475	(*E*)-Pentacos-10-ene	3.9[e]	tr[e]	tr[e]					+	MS, DMDS
2475	(*E*)-Pentacos-9-ene								+	MS, DMDS
2499	Docosan-1-ol						16.0	11.5	+	MS, RI, CoI, Si
2500	Pentacosane	6.7	0.8	tr	0.6				+	MS, RI, CoI
2516	Tricosan-2-one				tr				-	MS, RI
2521	Pentadecyl benzoate			tr					+	MS, RI, CoI
2525	Methyl docosanoate		tr	tr					+	MS, RI
2536	Tricosanal			tr	0.8				+	MS, RI
2540	(*Z*)-Hexacos-9-ene	0.4							-	MS, RI, DMDS
2563	2-Methylpentacosane	tr							+	MS, RI
2574	3-Methylpentacosane	0.5	tr						+	MS, RI
2577	Hexadecyl octanoate			tr					-	MS, RI
2593	Hexacos-1-ene	tr							+	MS, RI
2596	13-Methylpentadecyl benzoate		tr	tr					+	**NEW**
2600	Hexacosane	0.4	tr						+	MS, RI

2601	Tricosan-1-ol						0.6		-	MS, RI, Si
2612	Docosyl acetate			0.1	0.1				+	MS, RI
2617	Tetracosan-2-one				0.1				-	MS, RI
2625	Hexadecyl benzoate			0.1					+	MS, RI, CoI
2637	(*Z*)-Heptacos-10-ene	0.6[e]							+	MS, DMDS
2637	(*Z*)-Heptacos-9-ene								+	MS, DMDS
2638	Tetracosanal			0.2	1.2	2.2			+	MS, RI
2663	2-Methylhexacosane	0.9	tr						+	MS, RI
2673	Hexadecyl nonanoate			tr					-	MS, RI
2675	(*E*)-Heptacos-10-ene								+	MS, DMDS
2675	(*E*)-Heptacos-9-ene	2.2[e]	tr[e]						+	MS, DMDS
2675	(*E*)-Heptacos-8-ene								+	MS, DMDS
2686	2-Phenylethyl hexadecanoate			tr	0.1				-	MS, RI
2692	Heptacos-1-ene	1.0							-	MS, RI
2700	Heptacosane	7.2	0.8						+	MS, RI, CoI
2702	Tetracosan-1-ol						17.6	9.0	-	MS, RI, Si
2709	Tricosyl acetate			tr					-	MS, RI
2711	(*E*)-Heptacos-2-ene	0.3							+	MS, RI
2718	Pentacosan-2-one				2.2				+	MS, RI
2730	Methyl tetracosanoate			tr					+	MS, RI
2739	Pentacosanal			tr	0.5				+	MS, RI
2764	2-Methylheptacosane	tr							+	MS, RI
2768	Hexadecyl decanoate			0.2	0.1				-	MS, RI
2775	3-Methylheptacosane	1.3	tr						+	MS, RI

2778	(*E*)-Octacos-10-ene	0.2							-	MS, RI, DMDS
2790	Octacos-1-ene		tr						+	MS, RI
2800	Octacosane	0.6	tr						+	MS, RI, CoI
2805	Pentacosan-1-ol							tr	-	MS, RI, Si
2813	Tetracosyl acetate			0.3	0.4				+	MS, RI
2821	Hexacosan-2-one				0.2				+	MS, RI
2832	Octadecyl benzoate			tr					+	MS, RI, CoI
2839	(*Z*)-Nonacos-10-ene	0.5[e]							+	MS, DMDS
2839	(*Z*)-Nonacos-9-ene								-	MS, DMDS
2843	Hexacosanal			0.4	1.8				+	MS, RI
2864	2-Methyloctacosane	1.7	tr						+	MS, RI
2875	(*E*)-Nonacos-10-ene	4.1[e]	tr[e]						+	MS, DMDS
2875	(*E*)-Nonacos-9-ene								+	MS, DMDS
2890	2-Phenethyl octadecanoate				0.4				-	MS, RI
2900	Nonacosane	11.1	1.5	tr	0.4				+	MS, RI, CoI
2909	Hexacosan-1-ol					1.9	13.2	4.4	+	MS, RI, Si
2912	Pentacosyl acetate			tr					-	MS, RI
2925	Heptacosan-2-one				3.4				+	MS, RI
2934	Methyl hexacosanoate			0.1					+	MS, RI
2939	(*Z*)-Triacont-10-ene	0.5							-	MS, DMDS
2945	Heptacosanal			tr	0.4				+	MS, RI
2956	Tetradecyl tetradecanoate			0.7					+	MS, RI
2964	2-Methylnonacosane	2.8	tr						+	MS, RI
2969	Hexadecyl dodecanoate			0.3					+	MS, RI

2974	3-Methylnonacosane	1.0							+	MS, RI
2975	(*E*)-Triacont-10-ene	tr							+	MS, DMDS
2984	Hexyl docosanonate			tr					+	MS, RI
3000	Triacontane	1.3	tr						+	MS, RI, CoI
3010	Heptacosan-1-ol							tr	-	MS, RI, Si
3016	Hexacosyl acetate			0.2	0.2				+	MS, RI
3028	Octacosan-2-one				0.4				+	MS, RI
3040	Eicosyl benzoate			0.8					+	MS, RI, CoI
3042	(*Z*)-Hentriacont-10-ene	0.5	tr						+	MS, RI, DMDS
3049	Octacosanal			0.8	0.1	3.4			+	MS, RI
3063	2-Methyltriacontane	2.2	tr						+	MS, RI
3078	(*E*)-Hentriacont-10-ene	6.8[e]	0.6[e]						+	MS, RI, DMDS
3078	(*E*)-Hentriacont-9-ene								+	MS, RI, DMDS
3100	Hentriacontane	22.4	4.3	0.2	0.8				+	MS, RI
3114	Octacosan-1-ol						21.0	9.7	+	MS, RI, Si
3117	18-Methyleicosyl benzoate			tr					+	**NEW**
3130	Nonacosan-2-one				4.3				+	MS, RI
3140	Methyl octacosanoate			tr					+	MS, RI
3143	Heneicosyl benzoate			tr					+	MS, RI, CoI
3144	(*Z*)-Dotriacont-10-ene	0.4							+	MS, DMDS
3151	Tetradecyl hexadecanoate			1.9					+	MS, RI
3152	Nonacosanal				0.1				-	MS, RI
3152	Hexadecyl tetradecanoate			0.4					+	MS, RI
3165	Octadecyl dodecanoate			0.2					-	MS, RI

3173	3-Methylhentriacontane	2.3	tr						+	MS, RI
3197	Nonacosane-12,14-dione			4.1	2.6				+	MS, HZ, Si
3200	Dotriacontane	0.8	tr						+	MS, RI, CoI
3210	20-Methylheneicosyl benzoate			0.2					+	MS, RI
3215	Nonacosan-1-ol							tr	-	MS, RI, Si
3216	Octacosyl acetate			0.8	0.4				+	MS, RI
3232	Triacontan-2-one				0.2				+	MS, RI
3233	Docos-15-en-1-yl benzoate				0.2				+	MS, RI
3247	Docosyl benzoate			1.9					+	MS, RI, CoI
3254	Triacontanal			0.3	0.5				-	MS, RI
3264	2-Methyldotriacontane	0.1							+	MS, RI
3265	Pentadecyl hexadecanoate			0.1					-	MS, RI
3266	Hexadecyl pentadecanoate			0.2					-	MS, RI
3278	(*E*)-Tritriacont-10-ene	0.3[e]							+	MS, RI, DMDS
3282	(*E*)-Tritriacont-9-ene								+	MS, RI, DMDS
3295	Hentriacontan-16-one				tr	10.5			-	MS, RI
3297	Triacontane-14,16-dione			0.4	0.3				+	**NEW**
3300	Tritriacontane	1.7	tr						+	MS, RI, CoI
3316	Triacontan-1-ol					2.2	2.1		+	MS, RI, Si
3324	20-Methyldocosyl benzoate			tr					+	**NEW**
3334	Hentriacontan-2-one				0.9				+	MS, RI
3337	5α-Stigmasta-7,22-dien-3β-ol							13.6	+	MS, RI, Si
3350	Tricosyl benzoate			tr					+	MS, RI, CoI
3351	Tetradecyl octadecanoate			1.5					+	MS, RI

3352	Hexadecyl hexadecanoate		2.8			+	MS, RI
3365	2-Methyltritriacontane	0.1				-	MS, RI
3375	3-Methyltritriacontane	tr				-	MS, RI
3396	Hentriacontane-14,16-dione		55.3	45.6	32.6	+	MS, HZ, Si
3417	22-Methyltricosyl benzoate		tr			+	MS, RI
3427	21-Methyltricosyl benzoate		0.7			+	**NEW**
3453	Tetracosyl benzoate		1.1			+	MS, RI, CoI
3458	Dotriacontanal			0.5		-	MS, RI
3498	Dotriacontane-14,16-dione		0.7	0.5		+	**NEW**
3500	Pentatriacontane	0.2				+	MS, RI, CoI
3531	22-Methyltetracosyl benzoate		tr			+	**NEW**
3538	Tritriacontan-2-one			0.7		-	MS, RI
3556	Pentacosyl benzoate		tr			+	MS, RI
3572	Octadecyl hexadecanoate		0.4			-	MS, RI
3573	Hexadecyl octadecanoate		0.1			-	MS, RI
3598	Tritriacontan-16,18-dione		13.5	11.9		+	MS, HZ, Si
3610	Benzyl hexacosanoate		tr			+	MS, RI
3659	Benzyl 25-methylhexacosanoate		0.4			+	MS, RI
3660	Hexacosyl benzoate		0.1			+	MS, RI
3698	Tetratriacontane-16,18-dione		0.1	tr		+	**NEW**
3764	Hexadecyl eicosanoate		0.7			-	MS, RI
3765	Heptacosyl benzoate		0.5			+	MS, RI
3798	Pentatriacontane-16,18-dione		0.1	tr			MS, HZ
3870	Octacosyl benzoate		0.4			+	MS, RI

3950	Docosyl hexadecanoate			tr					+	MS, RI
3951	Eicosyl octadecanoate			tr					+	MS, RI
	**Total identified [%]**	**99.5**	**91.7**	**95.2**	**96.9**	**95.2**	**93.2**	**96.6**		

[a] RI – retention indices on a DB-5 column calculated against a series of co-injected *n*-alkanes for TMS derivatives; [b] Values are means of three individual analyses; tr, trace amounts (< 0.05%); [c] I = + denotes compounds identified in the *D. superbus* flower wax sample (Table 1), – denotes compounds detected only in chromatographic fractions. [d] ID = Compound identification: MS, mass spectra matching; RI, retention indices matching with literature data; CoI, co-injection with a pure reference compound; DMDS, identification by derivatization with dimethyl disulfide; HZ, identification by derivatization with hydrazine; Si, identification by derivatization with trimethylsilyl chloride; [e] Alkenes with different double bond locations represented one peak in the GC chromatogram and for that reason, it was not possible to determine their distinct relative amount in the washings.

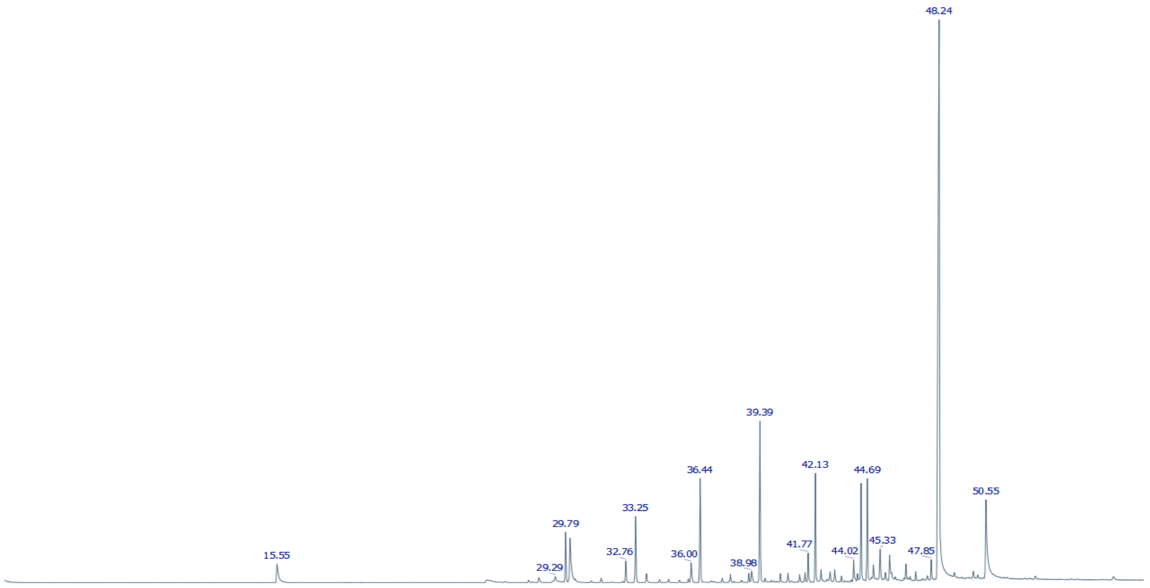

Figure S1. Typical total ion current (TIC) chromatogram of diethyl ether extract of *Dianthus petraeus* flowers (sample **5a** in Table 1).

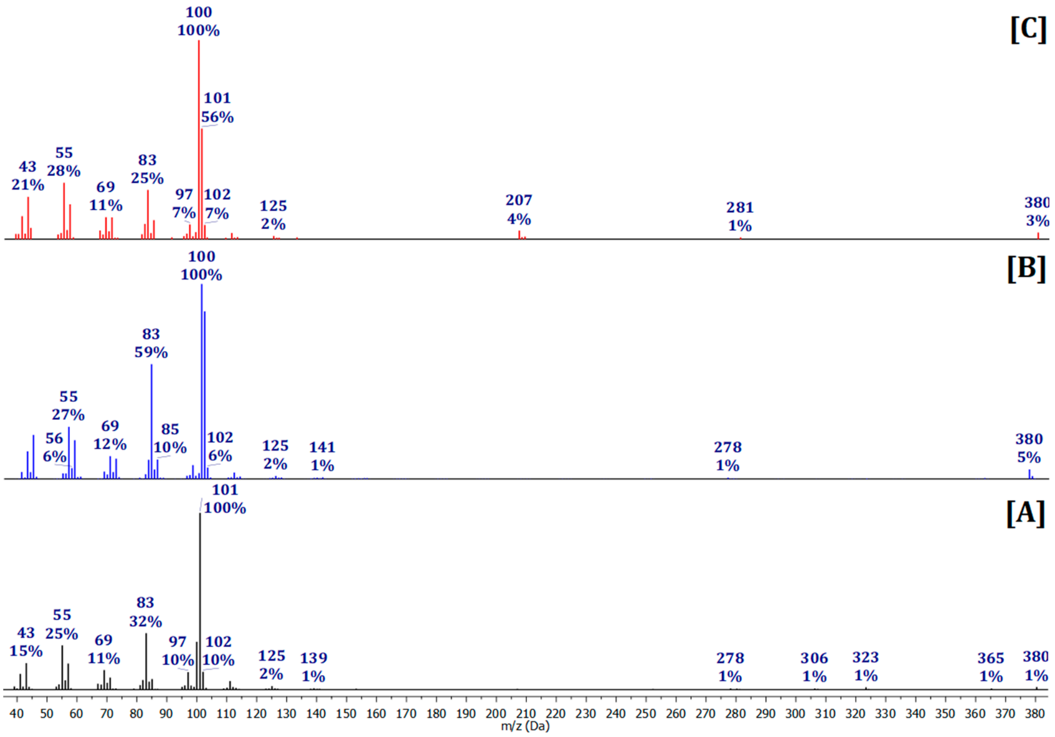

Figure S2. Mass spectrum of **[A]** eicosyl tiglate, **[B]** eicosyl senecioate, and **[C]** eicosyl angelate.

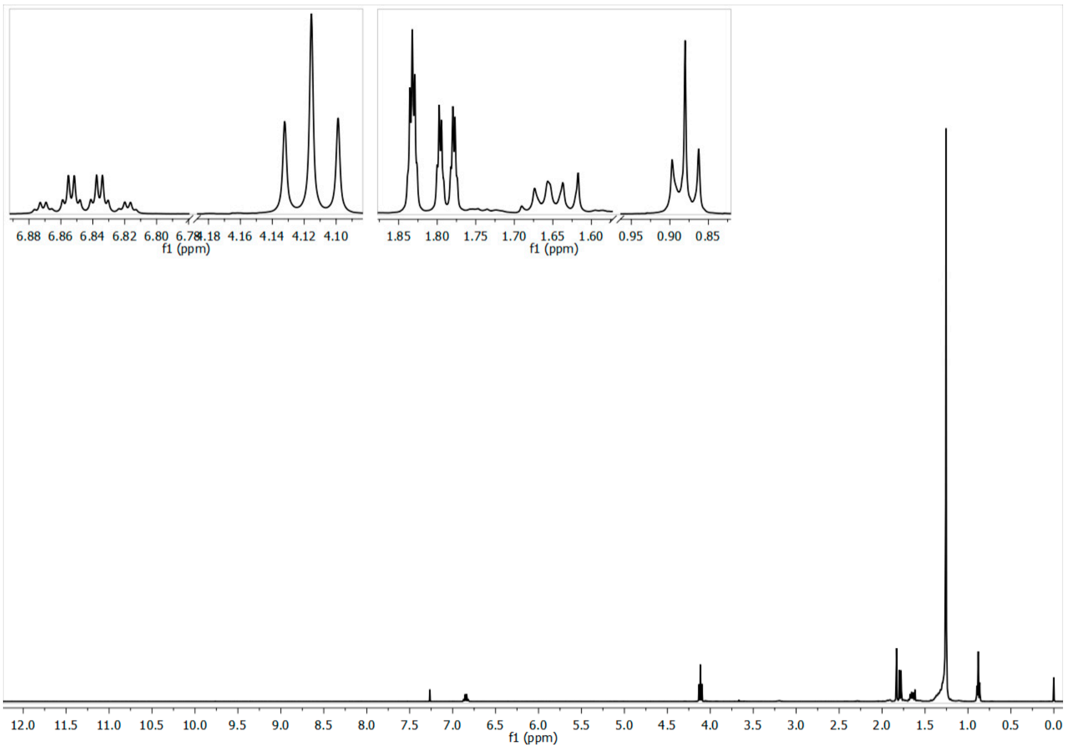

Figure S3. ^{1}H NMR spectrum of eicosyl tiglate recorded in $CDCl_3$.

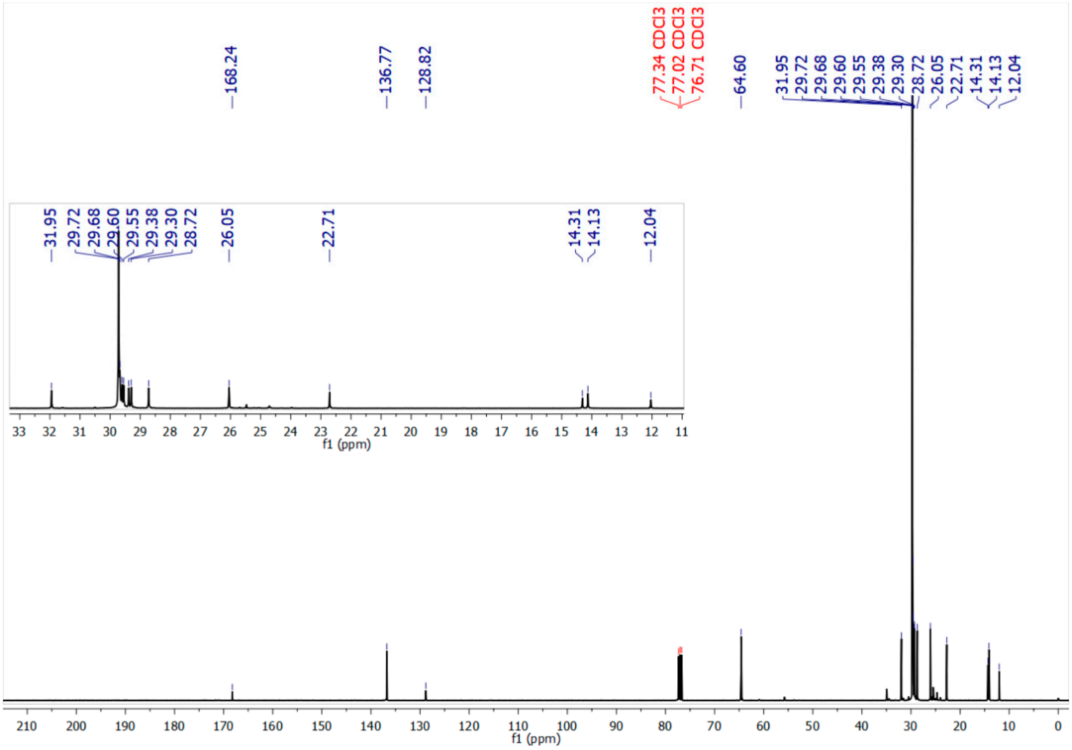

Figure S4. ^{13}C NMR spectrum of eicosyl tiglate recorded in $CDCl_3$.

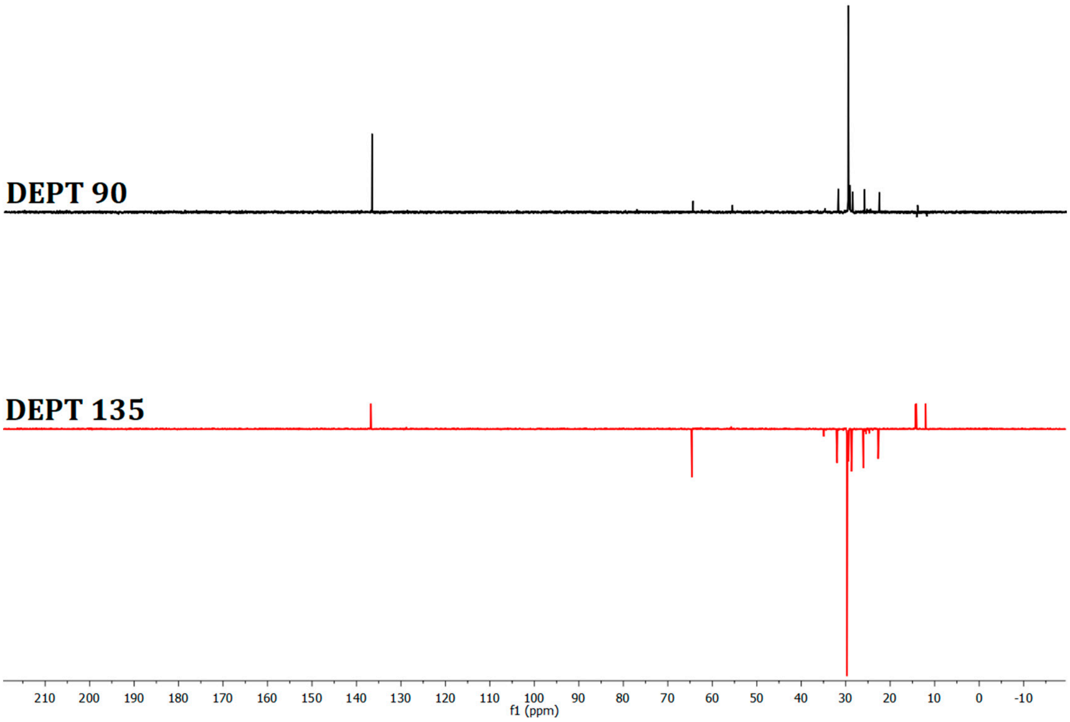

Figure S5. DEPT 90 and DEPT 135 spectra of eicosyl tiglate recorded in $CDCl_3$.

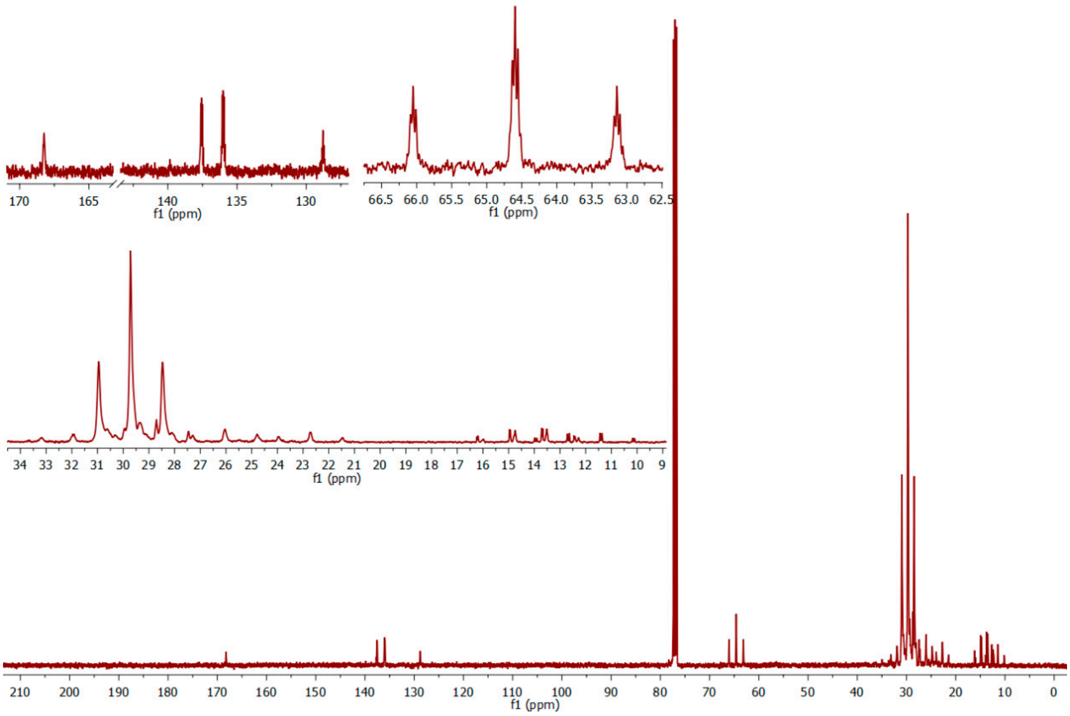

Figure S6. Proton-coupled ^{13}C NMR spectrum of eicosyl tiglate recorded in $CDCl_3$.

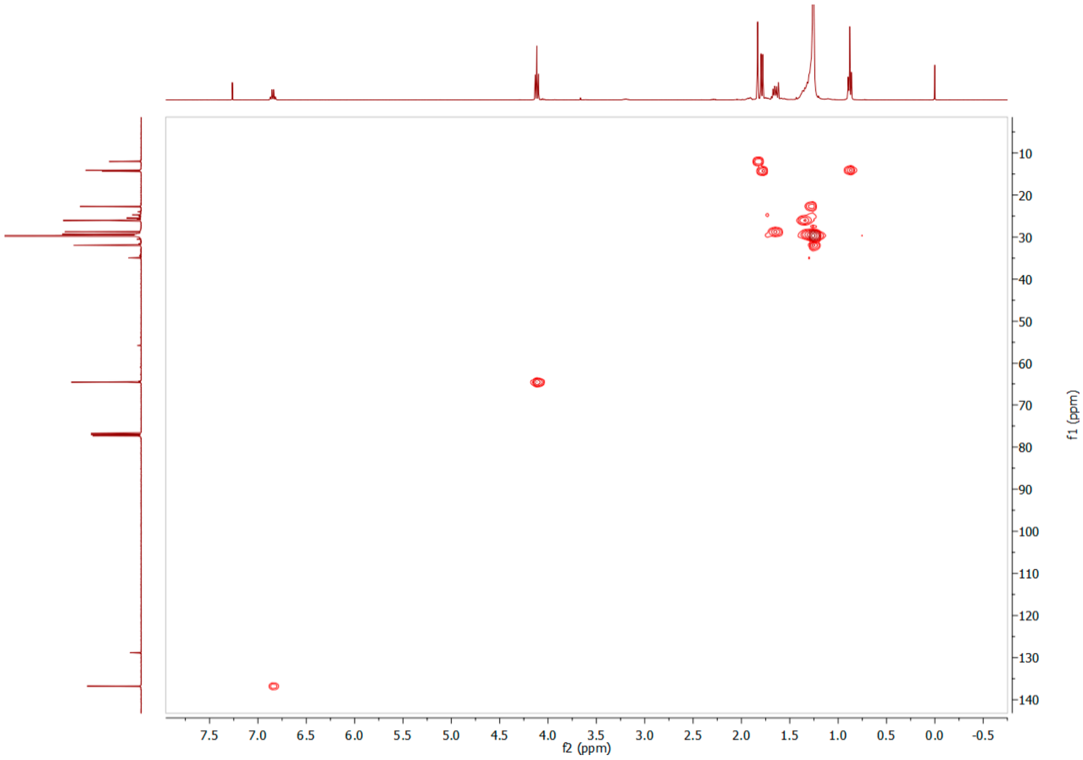

Figure S7. HSQC spectrum of eicosyl tiglate recorded in $CDCl_3$.

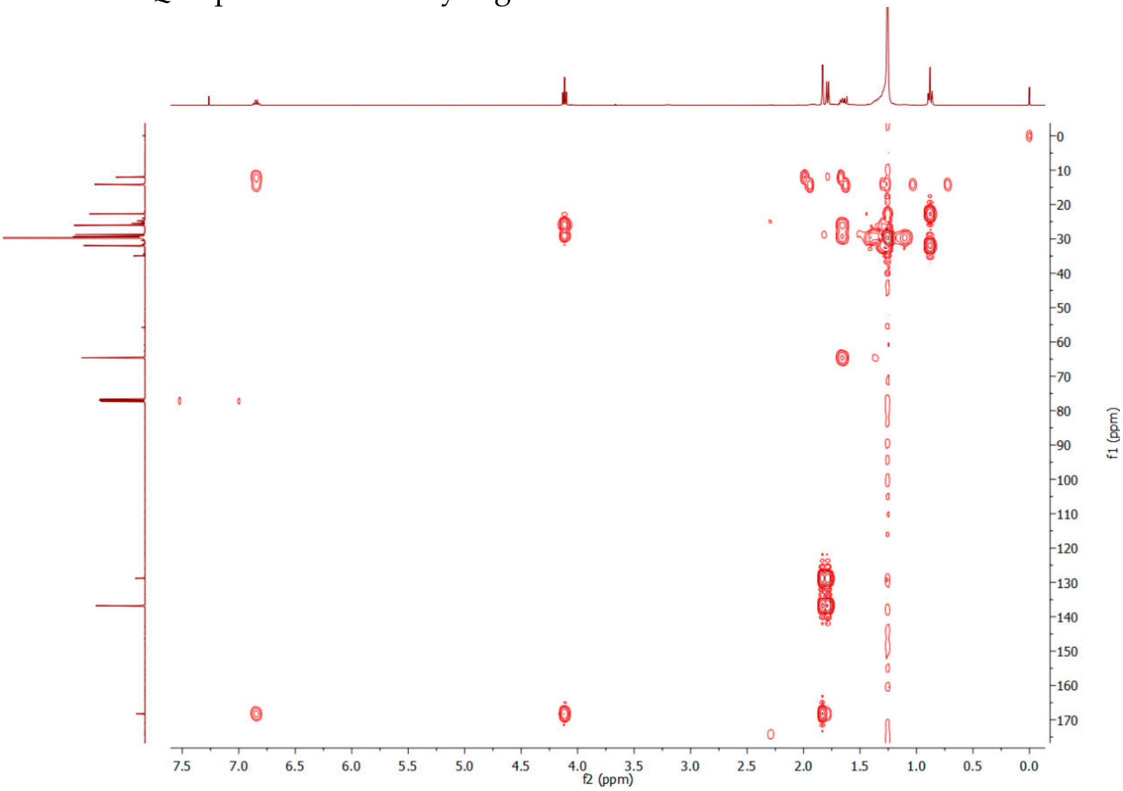

Figure S8. HMBC spectrum of eicosyl tiglate recorded in $CDCl_3$.

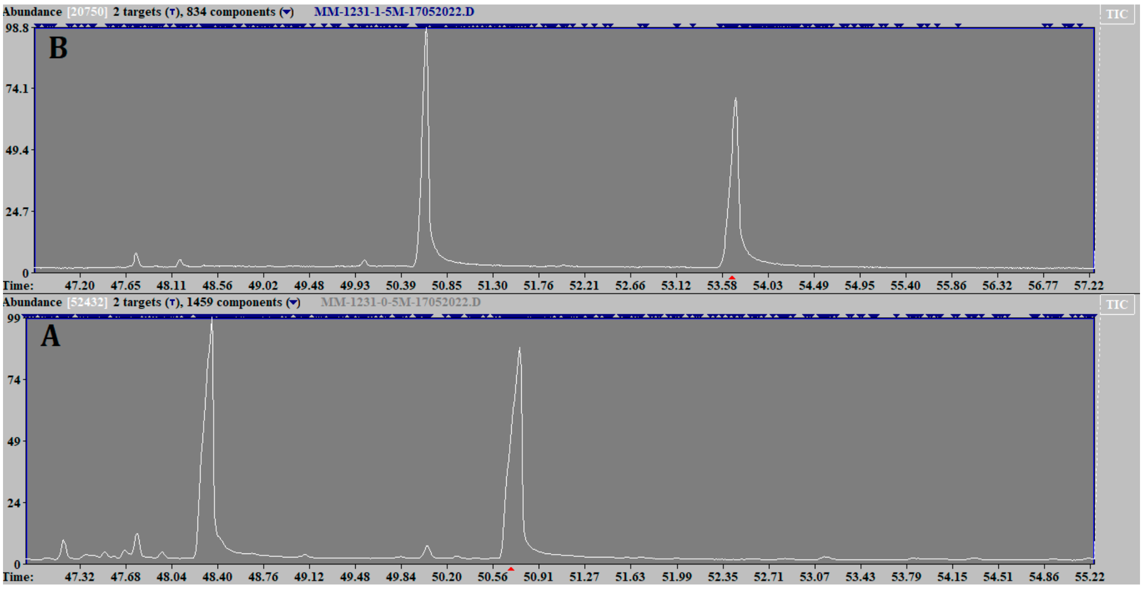

Figure S9. Part of the GC chromatogram before (**A**) and after (**B**) derivatization of crude wax sample with hydrazine.

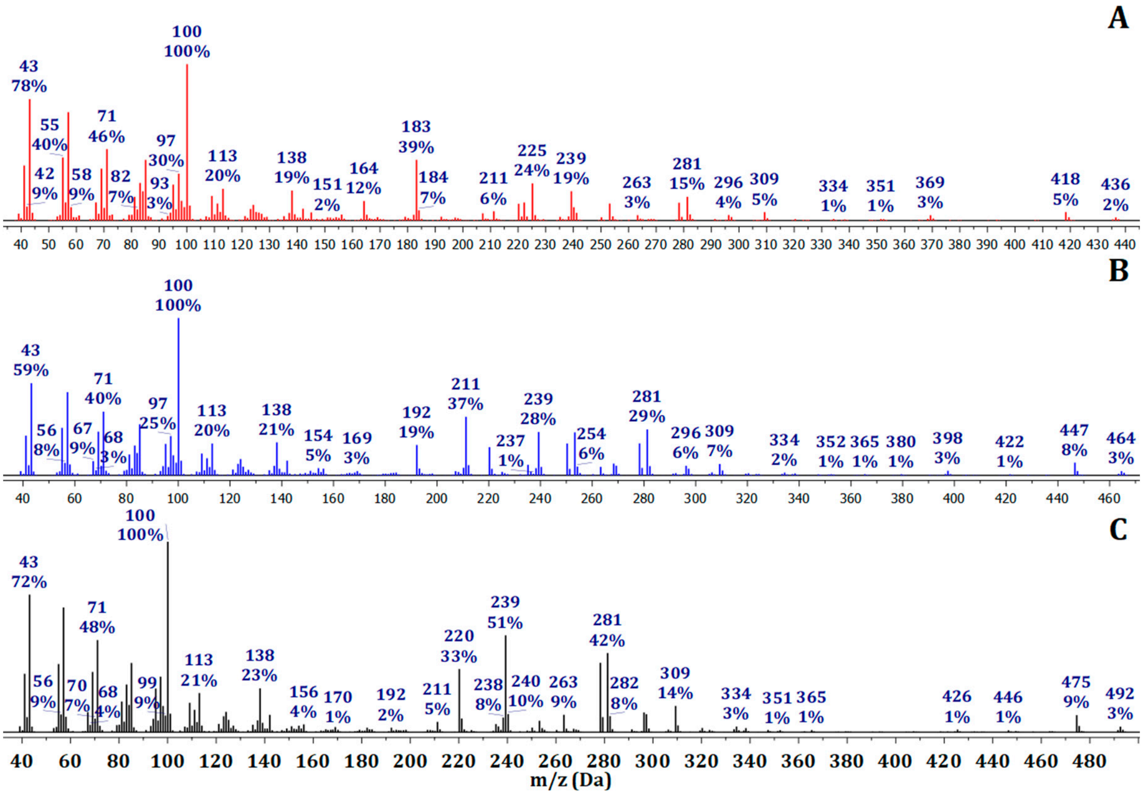

Figure S10. Mass spectra of nonacosane-12,14-dione (**A**), hentriacontane-14,16-dione (**B**), and tritriacontane-16,18-dione (**C**).

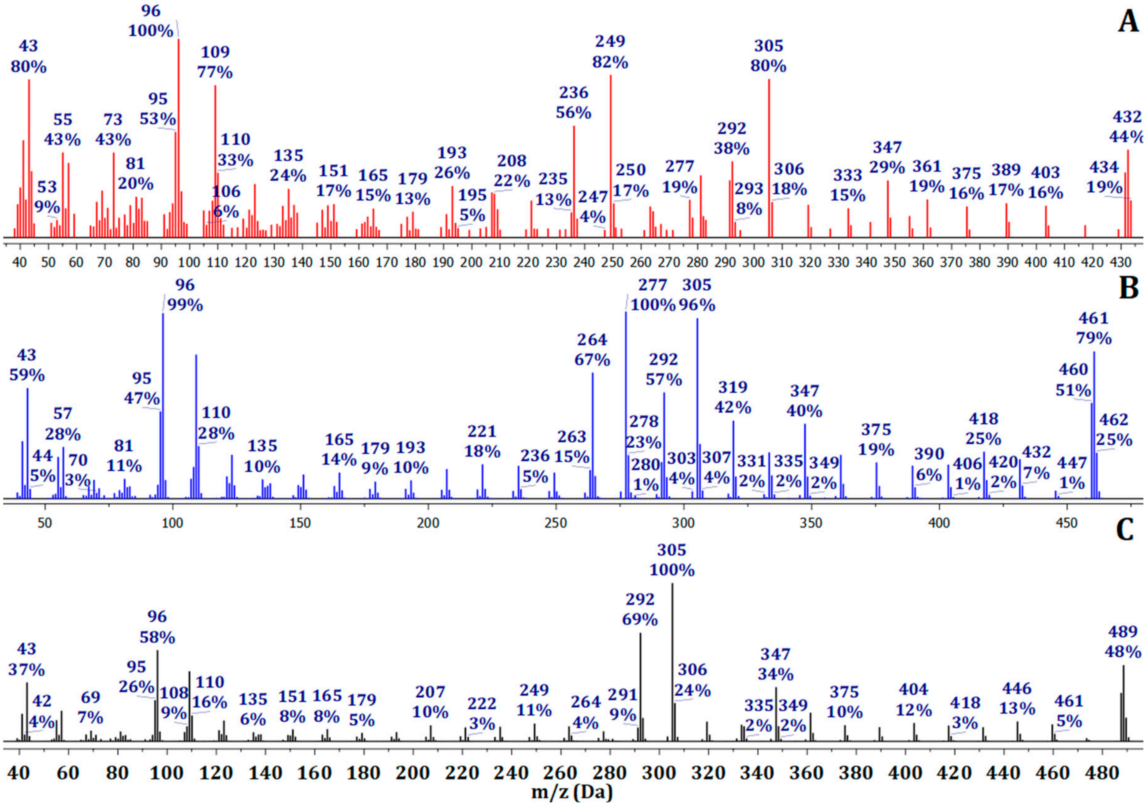

Figure S11. Mass spectra of pyrazole derivatives obtained from nonacosane-12,14-dione (**A**), hentriacontane-14,16-dione (**B**), and tritriacontane-16,18-dione (**C**).

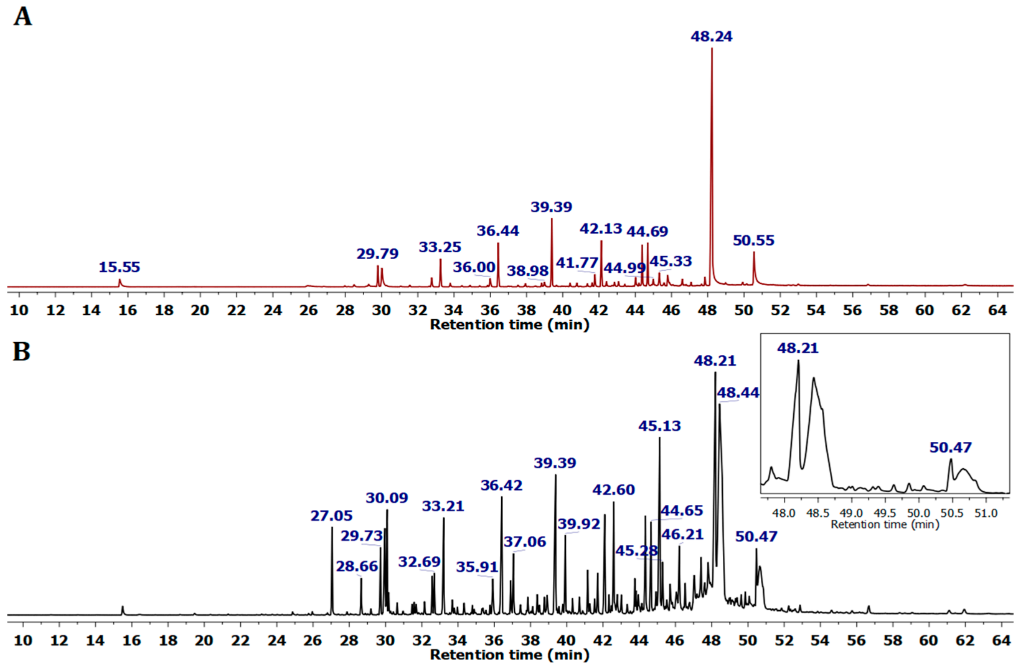

Figure S12. Part of the GC chromatogram before (**A**) and after (**B**) silylation of the crude wax sample.

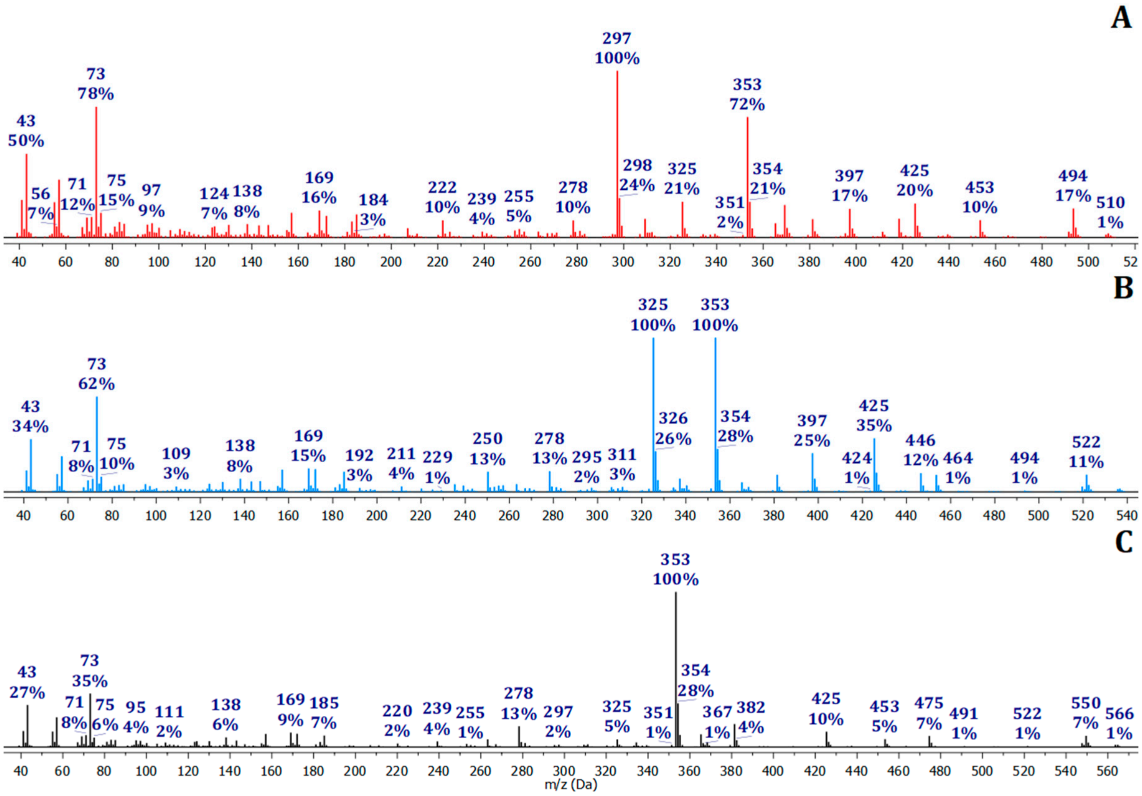

Figure S13. Mass spectra of mixtures of silylated enol forms of nonacosane-12,14-dione (**A**), hentriacontane-14,16-dione (**B**), and tritriacontane-16,18-dione (**C**).

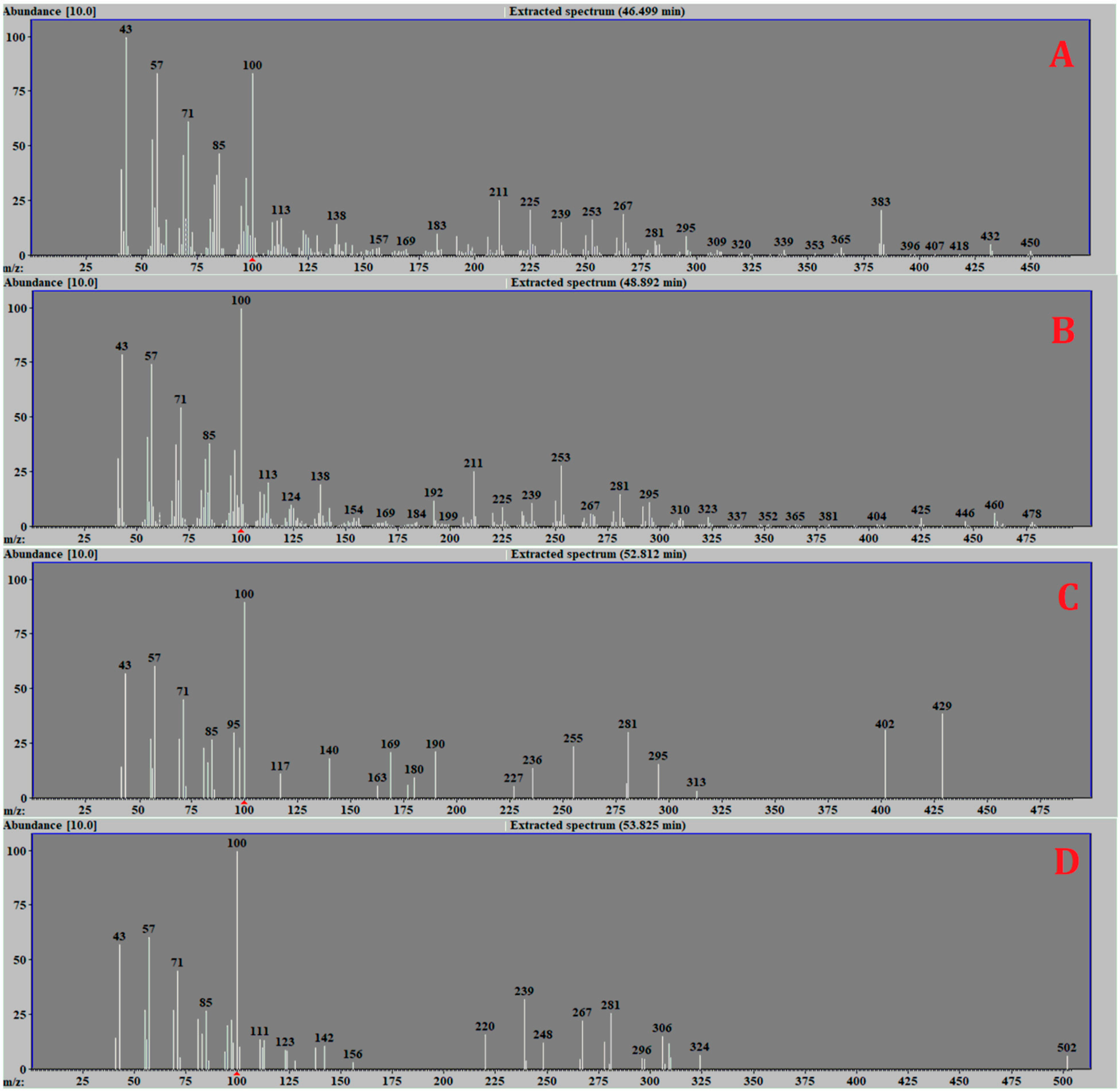

Figure S14. Mass spectra of triacontane-14,16-dione (**A**), dotriacontane-14,16-dione (**B**), tetratriacontane-16,18-dione (**C**), and pentatriacontane-16,18-dione (**D**).

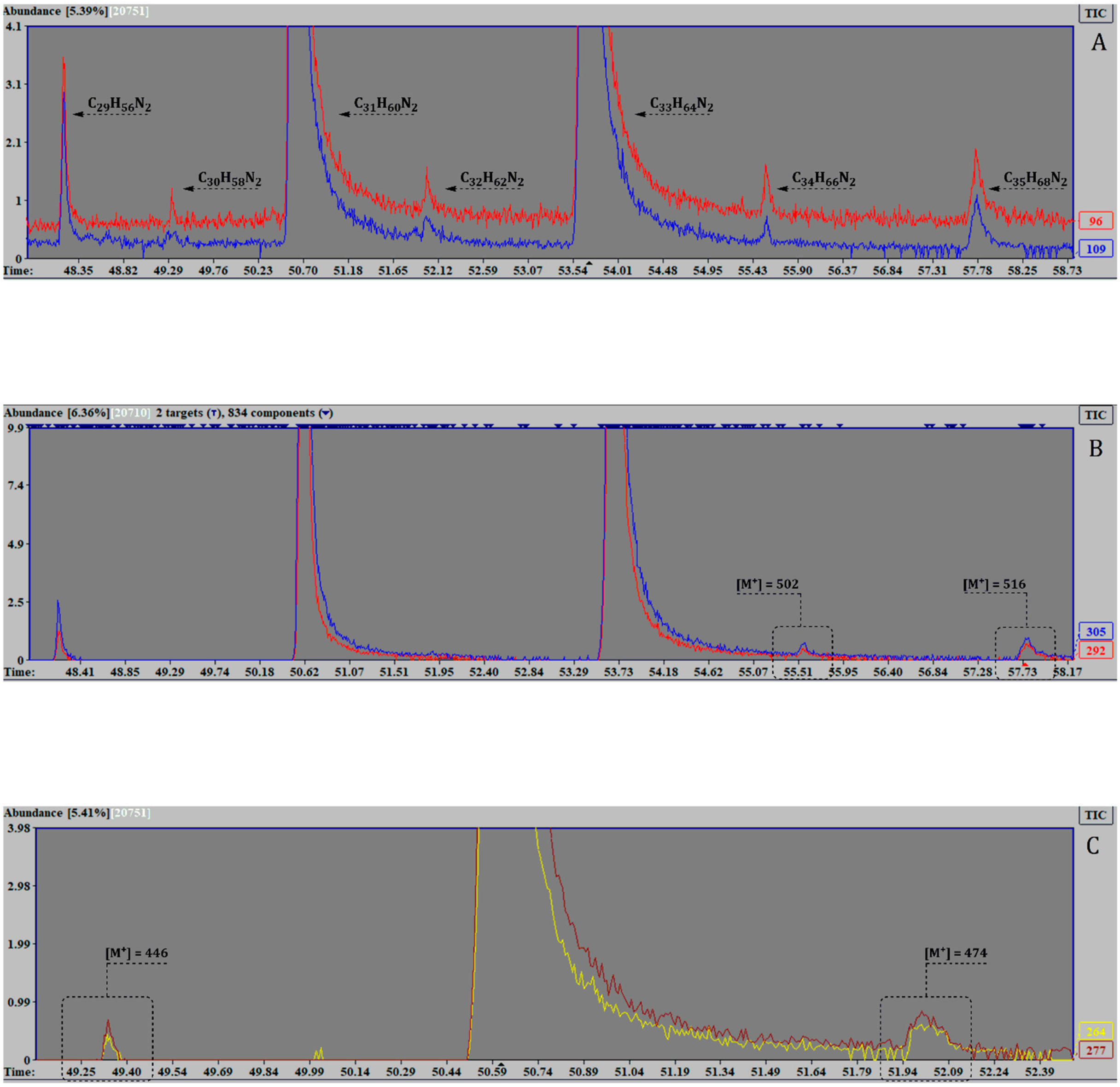

Figure S15. Patrial ion current chromatogram (PIC, ions at: **A**: *m*/*z* 96 and 109, **B**: *m*/*z* 292 and 305, and **C**: *m*/*z* 264 and 277) of derivatized chromatographic fraction of *D. superbus* flowers washings.

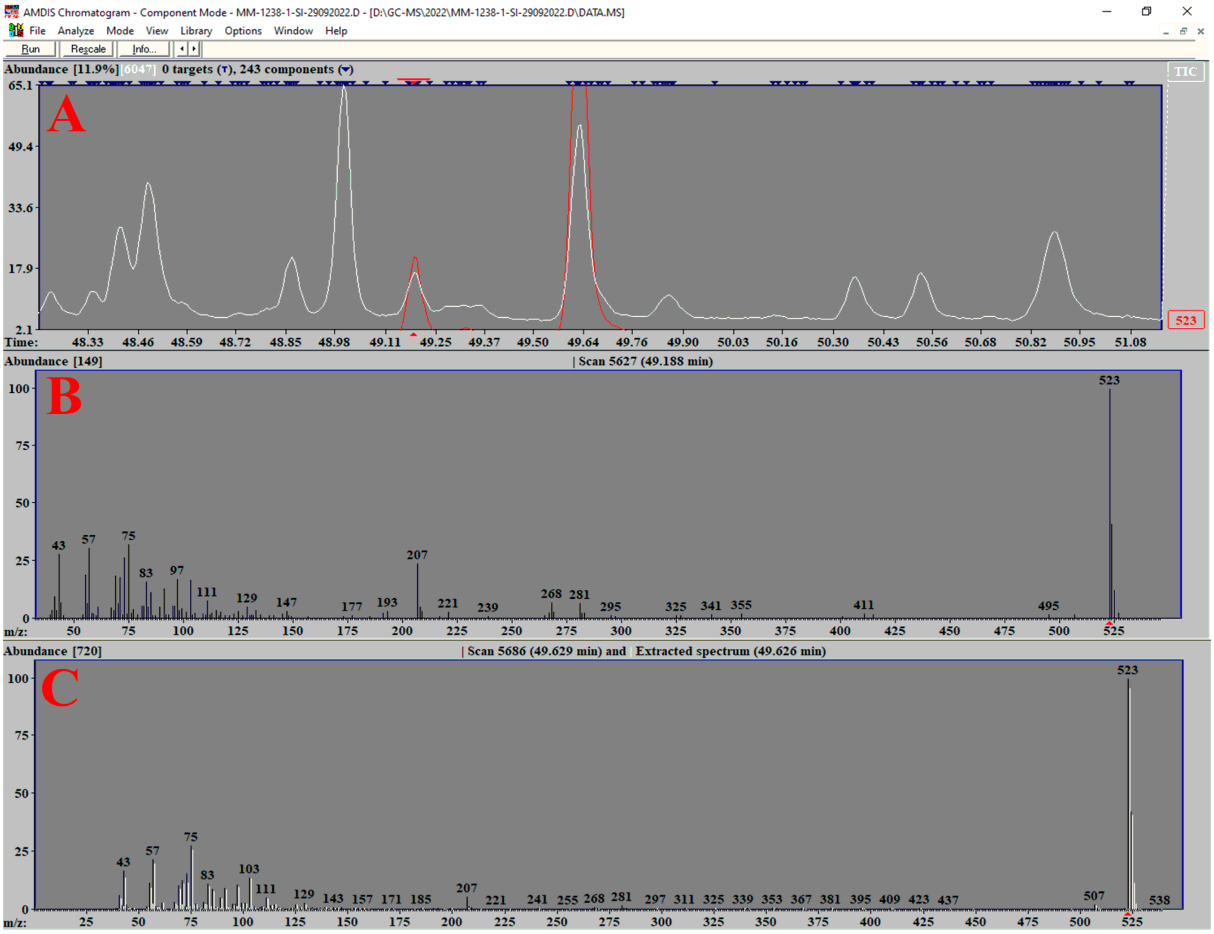

Figure S16. A - Part of the chromatogram (ca. 48 – 51 min) of the silylated wax sample; **B** - mass spectrum of unknown detected constituent at Rt = 49.15 min; **C** - mass spectrum of 1-(trimethylsilyloxy)dotriacontane detected at Rt = 49.56 min.

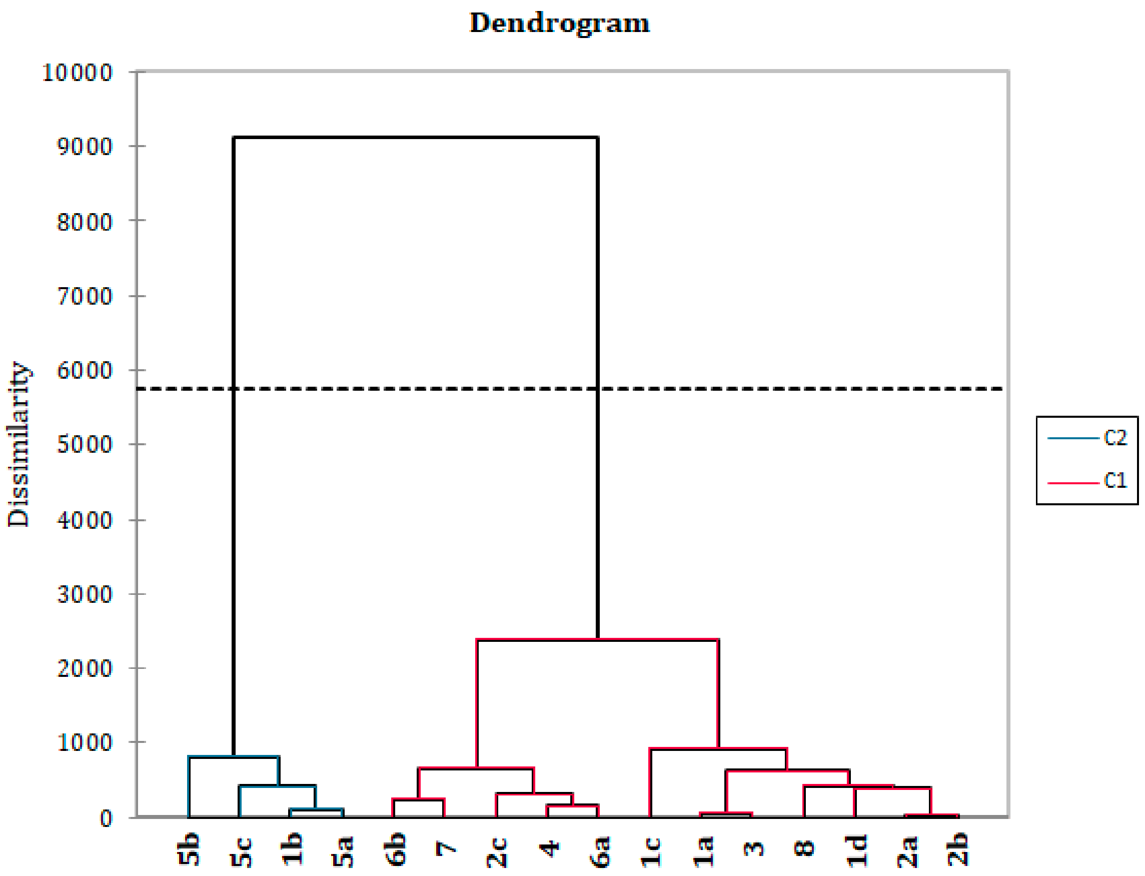

Figure S17. Dendrogram of AHC obtained by agglomerative hierarchical clustering using the transformed sums of constituent classes and representing the chemical-composition dissimilarity relationships of 16 wax samples (observations) of 7 different *Dianthus* taxa (15 samples) and one sample of *P. prolifera*. As a dissimilarity metric, the Euclidian distance was used (dissimilarity within the interval [0, 5800], using Ward's method as an aggregation criterion). Two statistically different groups of oils were found (**C1**–**C2**).

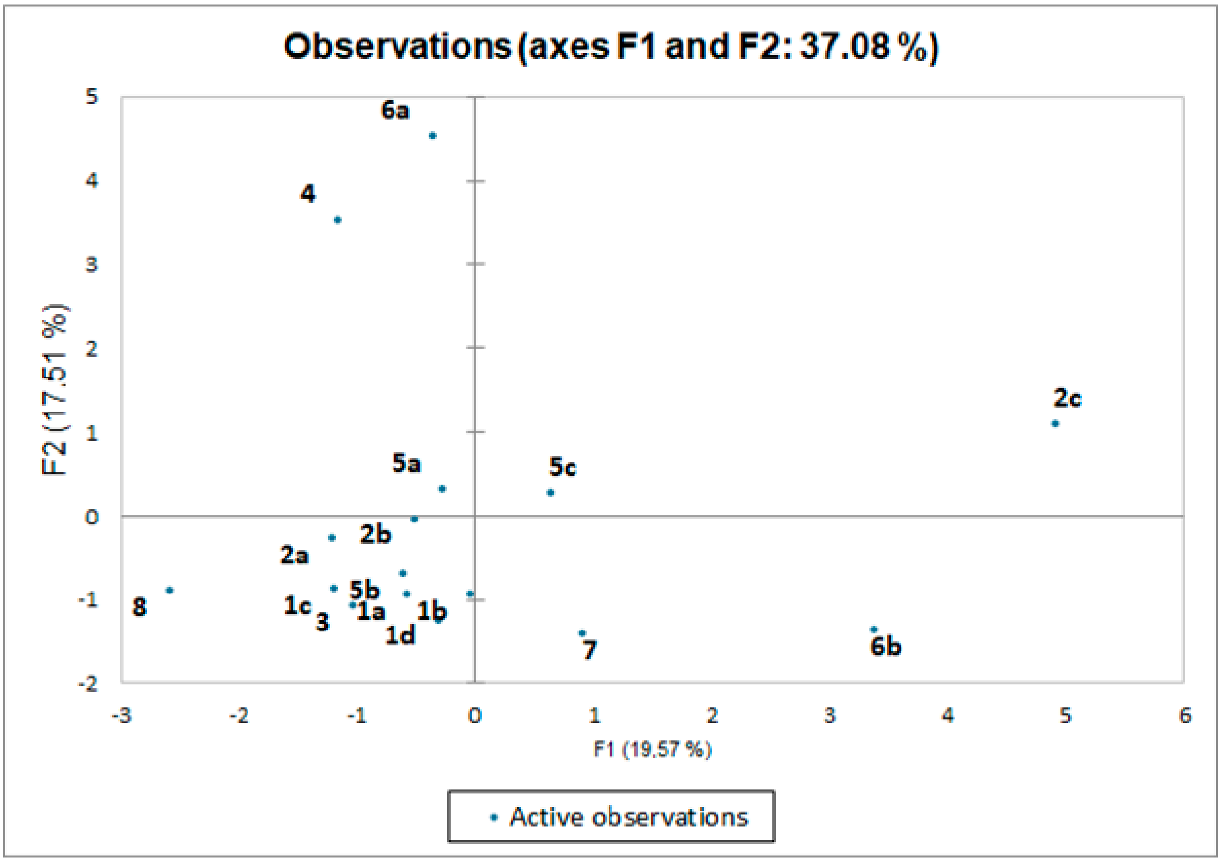

Figure S18. Dendrogram of PCA obtained by principal component analysis using the transformed sums of constituent classes and representing the chemical-composition dissimilarity relationships of 16 wax samples (observations) of 7 different *Dianthus* taxa (15 samples) and one sample of *P. prolifera*.

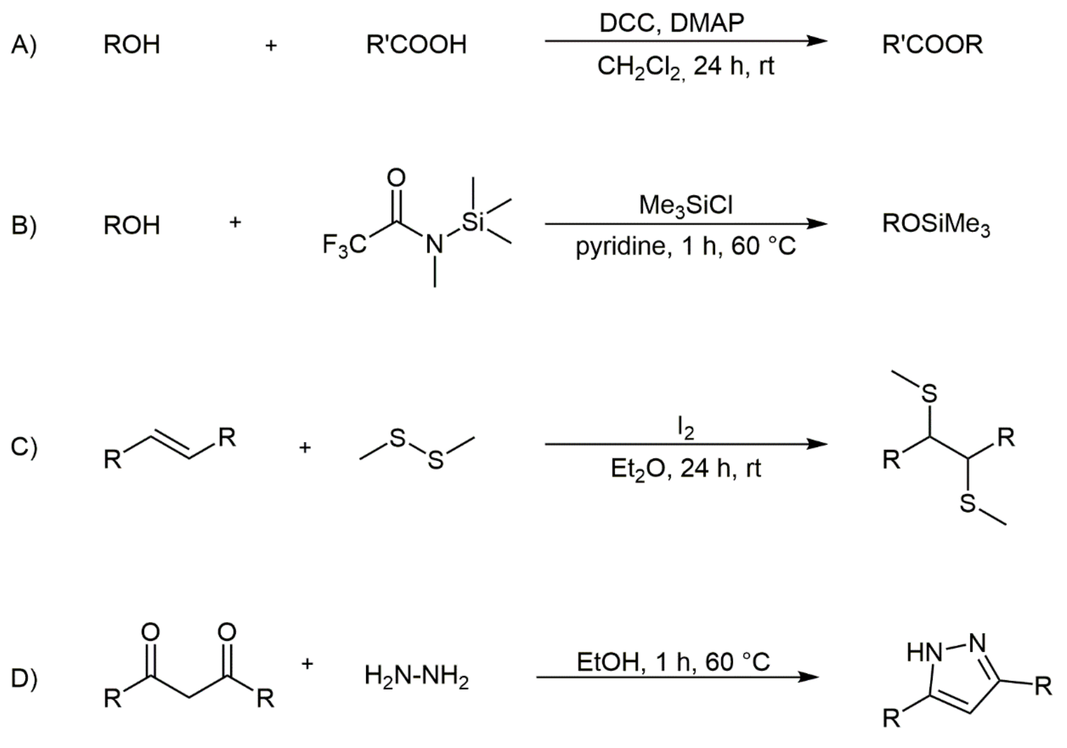

R, R' = alkyl chain

Figure S19. Esterification (A; DCC - *N*,*N'*-dicyclohexylcarbodiimide, DMAP - 4-(dimethylamino)pyridine), silylation (B), dimethyl disulfide (DMDS) derivatization (C), and the synthesis of pyrazoles (D).
